# The best exercises from top 20 by health-related indicators

**DOI:** 10.3389/fpubh.2025.1475618

**Published:** 2025-03-25

**Authors:** Albertas Skurvydas, Natalja Istomina, Dovilė Valanciene, Ruta Dadeliene, Ieva Egle Jamontaite, Ausra Lisinskiene, Asta Sarkauskiene, Daiva Majauskiene

**Affiliations:** ^1^Department of Rehabilitation, Physical and Sports Medicine, Institute of Health Sciences, Faculty of Medicine, Vilnius University, Vilnius, Lithuania; ^2^Faculty of Medicine, Institute of Health Sciences, Vilnius University, Vilnius, Lithuania; ^3^Faculty of Law, Vilnius University, Vilnius, Lithuania; ^4^Education Academy, Vytautas Magnus University, Kaunas, Lithuania; ^5^Departments of Sports Recreation and Tourism, Klaipėda University, Klaipėda, Lithuania

**Keywords:** athletes, behavior indicators, exercise, health indicators, physical activity

## Abstract

**Background:**

The study aimed to determine whether participation in professional sports, exercise in a sports/health center, or independent exercise (dependent variables) is associated with 11 health behavior-related indicators (independent variables) compared to having no exercise.

**Methods:**

The survey involved 293 professional Lithuanian athletes, 2,120 who exercise independently or in a sports/health centre and perform at least one of the 20 most popular exercise types in Lithuania (hereafter referred to as “E-20”), and 3,400 who do not exercise. The participants were aged 18–74 years.

**Results:**

The study uniquely examines a comprehensive range of 11 health-related indicators: body mass index, subjective health, depressed mood, stress, sedentary behavior, physical activity, sleep, alcohol consumption, smoking, overeating, and breakfast consumption. We examined whether these indicators differ between the three populations studied, whether they are associated with specific types of the E-20 exercises, and whether these patterns differ between men and women.

**Conclusion:**

Our study indicates that participants who engaged in physical activity generally scored higher on various health-related scales compared to those who were inactive. These benefits include reductions in depressed mood, stress, body mass index, and binge eating, as well as improvements in the regularity of breakfast consumption, vigorous physical activity, moderate to vigorous physical activity, and sleep duration (notably in men).

## Introduction

Research shows that different types and doses of exercise and physical activity effectively combat numerous chronic diseases (1, 2). Physical activity related health benefits are linked to health status, BMI, gender, and age (3–6). According to various studies, exercise and physical activity affect the body in specific manner; i.e., the effects depend on the intensity, motor control, duration, muscle contraction type, and other factors (7–11). In addition to physical activity, alcohol consumption, eating habits and smoking are also important determinants of a healthy lifestyle (12–16). Our recent research shows that physical activity enhances emotional health more than logical thinking (17, 18). Furthermore, the well-being benefits of physical activity are greater when it is mainly practiced during leisure time (19, 20).

Research has focused on how different indicators of human health are affected by various types of exercise and physical activity. Berger and Owen (7) studied how the four modes of exercise in different sports such as hatha yoga, fencing, swimming, and body conditioning reduce stress and improve mood. The authors reported that, whereas all exercise modes improved mood and reduced stress, fencing was the most beneficial. Matias et al. (11) found that depression was reduced by walking, cycling, running, or team sports. Wennman and Borodulin (21) investigated the associations between the type of physical activity, such as cleaning, gardening, walking, stair climbing, jogging, swimming, skiing, and achieving the physical activity recommendations. Chekroud et al. (9) analysed the data of 1.2 million people and investigated the link between physical exercise (recreation, aerobics or gym activities, water sports, jogging or running, cycling, walking, household chores, popular sports, and winter sports) and mental health. They determined that cycling and popular (team) sports had the lowest burden on mental health (9). Schnohr et al. (8) reported that life expectancy gains were associated with participation in sport and physical activity compared with being sedentary.

Despite the existing body of research, there is a notable lack of studies examining the association between health-related indicators and the most popular types of exercise engaged in for active recreation or health improvement. Our study aimed to determine whether 11 health behavior-related indicators are linked to independent exercise, professional sports, or exercise within a sports or health club, in contrast to individuals who do not exercise at all. We ranked the top 20 exercise types in Lithuania (E-20) that are not performed by professional athletes but are practiced by individuals who exercise independently or at a sports/health center. We then compared this ranking with that of professional athletes and those who are physically inactive—defined as individuals who do not engage in any form of physical exercise. The E-20 exercises were evaluated based on their potential benefits for various health-related indicators including BMI, subjective health, depressed mood, stress, sedentary behavior, physical activity levels, sleep quality, alcohol consumption, smoking habits, overeating, and breakfast consumption.

## Materials and methods

### Participants

We surveyed 293 professional Lithuanian athletes, 2,120 people who exercised independently or in health/sports clubs and did one or some E-20 exercises, and 3,400 people who did not exercise at all (Table 1). The age of the respondents ranged from 18 to 74 years (Table 1). The study was carried out from October 2019 to June 2020. The respondents were chosen from the entire country to represent the Lithuanian population. The data was collected through the online survey created using Google Forms (22). Google Forms. Google. https://docs.google.com/forms/) and distributed via a personal messaging app (WhatsApp) and social media. Measures were taken to ensure the anonymity of the participants and the confidentiality of the data collected and processed. Data of the participants who completed the online questionnaire were used for the study.

**Table 1:** Descriptive characteristics of subjects and Health-related indicators.

	Sex	I do not exercise		I do professional sport		I exercise “E-20″		Effect of exercise, *p* value	Effect of gender, p value	Interaction effect, exercise x gender	Test used
Descriptive characteristics of subjects											
Count	W*	1726		154		2,324					
	M**	394		139		1,176					
Age, years	W	39_b_	11.8	29.1_a_	10.5	38.3_b_	10.3				ANOVA
	M	37.7_b_	11.1	28.7_a_	9.3	35.7_c_	9.1				
Exercise experience, years	W	0		9.5_a_	7.7	6.1_b_	7.6				
	M	0		12_a_	8.1	8.8_b_	6.1				
Total types of exercises		0		44		20	0				
Subjective indicators											
Health	W	2.91	0.69	2.93	0.71	2.92	0.74	0.64	0.74	0.68	ANOVA, Tukey post
	M	2.95	0.68	2.88	0.73	2.93	0.71				
Depressed mood	W	2.13a	0.97	1.96	0.92	2.01	0.82	<0.001	<0.001	0.28	
	M	2.02_a_	0.97	1.75	0.95	1.81	0.81				
Stress	W	18.8_a_	6.5	17.42	6.2	17.36	6.7	<0.001	<0.001	0.085	
	M	18.4_a_	6.1	16.2	5.8	16.9	6.5				
Body mass index											
BMI, kg/m^2^	W	25.2_a_	5.2	22.3_b_	3.1	23.4_c_	3.3	<0.001	<0.001	0.18	ANOVA
	M	26.7_a_	4.1	24.8_b_	3.5	25.5_c_	3.1				
Energy expenditure											
Sleep, METs	W	6.55	0.79	6.84_a_	0.86	6.6	0.9	<0.001	0.059	0.04	ANOVA
	M	6.23_a_	0.94	6.85_b_	0.91	6.51_c_	0.92				
SB***, METs	W	14.5_a_	3.8	11.6_b_	3.8	13.4_c_	3.4	<0.001	0.002	0.47	
	M	15.2_a_	3.9	11.8_b_	3.9	13.5_c_	3.1				
LPA***, METs	W	8.2	4.1	6.64_a_	3.1	7.9	3.5	0.014	0.001	0.49	
	M	6.35	3.4	5.49_a_	3.2	6.05	3.1				
MPA***, METs	W	6.08	3.9	5.94	5.1	6.45_a_	5.7	0.048	0.25	0.74	
	M	6.01	4.8	5.89	4.9	6.37_a_	5.3				
VPA***, METs	W	1.7a	3.6	18.1_b_	10.1	8.9c	7.1	<0.001	<0.001	0.19	
	M	3.3a	6.4	19.8_b_	9.2	11.9_c_	8.1				
MVPA***, METs	W	7.78_a_	7.4	23.4_b_	12.9	14.4_c_	10.1	<0.001	<0.001	0.27	
	M	9.3a	11.2	25b	11.2	17.1_c_	11.2				
TEE***, METs	W	36.9_a_	7.1	48.5_b_	10.2	42.7_c_	7.3	<0.001	0.009	0.13	
	M	37.1_a_	8.4	49.3_b_	8.8	43.5_c_	7.7				
Healthy eating, smoking, and drinking											
Breakfast	W	2.48_a_	0.77	2.73	0.41	2.6	0.44	<0.001	0.37	0.19	
	M	2.37_a_	0.67	2.69	0.42	2.64	0.34				
Overeating	W	1.3a	0.61	0.93	0.62	0.97	0.54	<0.001	0.91	0.23	
	M	1.18_a_	0.61	0.94	0.4	0.96	0.38				
Smoking	W	1.97	1.2	1.95	1.2	1.98	1.1	0.028	<0.001	0.049	
	M	2.42	1.3	1.99_a_	1.2	2.31	1.2				
Alcohol drinking	W	3.19	1.7	3.14	1.7	3.24	1.5	<0.001	<0.001	<0.001	
	M	3.84_a_	1.4	2.95_b_	1.3	3.42_c_	1.1				

The matching letters indicate no significant difference between groups, and non-matching letters indicate significant differences (*p* < 0.05). *W–Women; ** M–Men. SB – sedentary behavior, LPA – light-intensity physical activity, MPA – moderate-intensity physical activity, VPA – vigorous-intensity physical activity, MVPA– moderate-to-vigorous intensity physical activity, TEE – total energy expenditure.

Klaipeda University Ethics Committee provided the approval to conduct this research (protocol No. STIMC-BTMEK-08) and affirmed that our research abided by the ethical standards of the Declaration of Helsinki (Revised 2013) and the National guidelines for biomedical and health research involving human participants (2017).

From the survey, we determined the age, body mass index (BMI), exercise experience and specific physical activity (PA) used by the participants, who were professional athletes, sedentary people, and athletic people exercising in sports and health centers or independently. The prevalent sports among professional athletes were as follows: track-and-field athletics (*n* = 30; both male and female), sports dancing (*n* = 25), volleyball (*n* = 24), basketball (*n* = 24), karate (*n* = 20), fitness (*n* = 19), running (*n* = 18), football (*n* = 17), roller skating (*n* = 14), martial arts (*n* = 14), powerlifting (*n* = 12), wrestling (*n* = 12), tennis (*n* = 10), handball (*n* = 9), boxing (*n* = 9), swimming (*n* = 9), rowing (*n* = 6) extreme conditioning program training (*n* = 6), aerobic (*n* = 6), bodybuilding (*n* = 5), triathlon (*n* = 5), cycling (*n* = 3). The 20 most popular exercises (sports disciplines) practiced by individuals focusing on strengthening their health are presented in Table 2.

**Table 2:** Top 20 exercises for men and women.

Men’s top 20 exercise	Men (Count)	Women’s top 20 exercise	Women (Count)
Basketball	161	Exercise/ independent	564
Running	152	Yoga	242
Resistance exercise	126	Resistance exercise	181
Exercise/ independent	113	Running	176
Cycling	73	Pilates	162
Martial arts	70	Aerobic	142
Football	61	Fitness	136
ECPT*	58	Dance	127
Volleyball	48	Callanetics	77
Fitness	42	Swimming	69
Swimming	41	Gym	64
Body building	38	Volleyball	63
Tennis	35	Track and field athletic	56
Track and field athletic	26	Tennis	54
Boxing	25	Cycling	48
Wrestling	24	Martial arts	48
Gym	22	ECPT*	38
Yoga	21	Basketball	32
Triathlon	20	Stretching	30
Power lifting	20	Cardio	15

ECPT*- extreme conditioning program training.

### Measurements

The *Danish Physical Activity Questionnaire (DPAQ)* adapted from the *International Physical Activity Questionnaire (IPAQ)* was used. In contrast to measuring PA in the past 7 days, the participants were asked to report their PA in the past 24 h for 7 consecutive days. The reported activities were noted on the PA scale and rated by metabolic equivalents (METs) at nine levels of physical exertion, ranging from sleep or inactivity (0.9 MET) to vigorous intensity physical activities (>6 METs). MET levels were marked in letters and illustrated by small drawings (*A* = 0.9 MET, B = 1.0 MET, *C* = 1.5 METs, *D* = 2.0 METs, *E* = 3.0 METs, *F* = 4.0 METs, *G* = 5.0 METs, *H* = 6.0 METs, and I > 6 METs). The participants had to mark on the scale the number of minutes (15, 30, or 45) and hours (1–10) spent for a particular PA at each MET level on an average weekday (24 h). The total MET time, including sleep, work, and recreation on an average weekday, was calculated (23). We determined how much energy (in METs) was spent per day during sleep, sedentary behavior, light intensity PA, moderate-intensity PA, and vigorous-intensity PA. MPA was combined with VPA as moderate-to-vigorous PA (MVPA) and the total energy expenditure (TEE) during 24 h was determined.

The *Subjective health assessment* was conducted using a four-point Likert-type scale, where participants were asked to rate their overall health. The scale was designed to capture a range of self-reported health statuses, from poor to excellent, with the following categories: 1 point: Poor health; 2 points: Satisfactory health; 3 points: Good health; 4 points: Excellent health. The four categories represent a spectrum of perceived health status, which is used in health research to capture individual perceptions of health.

Perceived stress was assessed using the *10-item Perceived Stress Scale (PSS-10).* The *PSS-10* is a 10-item self-reported questionnaire designed to assess the extent to which the individual has perceived situations in their life as unpredictable, uncontrollable and overloading over the past month. It consists of 10 questions, which are designed to be answered on a five-point scale ranging from *0 = never* to *4 = very often* on the Likert scale. Total scores ranged from 0 to 40, with higher scores indicating a higher perceived stress level (24). Moderate PSS is defined as a score ranging from 14 to 26, with scores below 14 indicating low stress and scores above 27 indicating high stress. The Perceived Stress Scale has dem demonstrated high internal consistency, with Cronbach’s alpha values typically ranging from 0.78 to 0.91.

In order to assess the manifestation of *Depressed mood*, a separate question was used in this study. The depressed mood was measured using a four-point Likert-type scale, where participants were asked to rate how frequently they have experienced a depressed mood recently. Response options were provided in the following order: *Not at all*, *No more than usual*, *Slightly more than usual*, *Much more than usual*. Answers were coded from 1 to 4, with a higher score indicating a greater frequency of depressed mood. This question was used as an additional indicator to preliminarily assess the occurrence of depressed mood.

*Smoking and alcohol consumption.* The respondents had to indicate their smoking habits on a scale of 1 to 4, where 1 is “I have never smoked”; 2 is “I smoke occasionally”; 3 is “I smoke every day”; 4 is “I used to smoke, but quit.” Alcohol consumption was assessed on a scale of 1 to 7, where 1 is “I do not drink at all”; 2 is “Several times a year”; 3 is “Once a month”; 4 is “Several times a month”; 5 is “Once a week”; 6 is “Several times a week”; and 7 is “Daily.”

*Eating Breakfast*. Eating breakfast was assessed on a three-point scale: 1 – No (I do not eat breakfast); 2 – Sometimes (I eat breakfast occasionally); 3 – Yes (I eat breakfast regularly).This scale was chosen to measure the frequency of breakfast consumption and to assess how regularly participants engage in this habit. We aimed to capture variations in breakfast eating behavior in a straightforward manner, considering both habitual eaters and those who eat breakfast infrequently or never.

*Overeating*. Overeating was also assessed using a similar three-point scale: 1 – No (I do not overeating); 2 – Rarely (I overeat on rare occasions); 3 – Often (I often overeat). The goal of this scale was to evaluate the frequency of overeating behavior, as overeating can have significant health implications.

### Statistical analysis

The interval data are reported as the mean ± standard deviation. Before conducting ANOVA, the assumptions of normality and homogeneity of variances were tested. The Shapiro–Wilk test was used to assess the normality of the data distribution, and Levene’s test was applied to evaluate the homogeneity of variances. If the assumptions were violated, appropriate adjustments or non-parametric tests were considered. The effect of independent variables (exercising among professional athletes, E-20, sedentary behavior) (gender: female, male) on the dependent variables (BMI, subjective health, perceived stress, depressed mood, sleep, SB, LPA, MPA, VPA, MVPA, TEE, smoking, alcohol consumption, eating breakfast, overeating) was determined by running a two-way ANOVA (Table 1). To reject the null hypothesis, the *p* value was defined <0.05. The partial eta squared $({ŋ}_{P}^{2}$ ) value was calculated the see the effect of independent variables on dependent variables. If significant effects were found, a *post hoc* test using the Tukey’s method was used for multiple comparisons within each repeated measures of ANOVA. Statistics software IBM SPSS statistics for Windows, Version 22.0 was used for data analysis and hypothesis testing (25). We also calculated the percentage of women and men who met several specific criteria: a normal body mass index (BMI) of 18.5–25 kg/m^2^, excellent health, no signs of depressed mood, low perceived stress (belonging to the lowest third of participants in terms of stress), non-smokers, no alcohol consumption, regular breakfast consumption, and avoidance of overeating. These calculations were performed for participants involved in E-20, which includes the 20 most popular sports in Lithuania. This group consists of non-professional athletes, such as individuals who attend sports and health clubs or engage in independent exercise, as well as professional athletes and those who are completely physically inactive The chi-square (χ2) and its *p* value were calculated in all cases, separately for men and women. We ranked 20 top exercise types in Lithuania (E-20) that are not practiced by professional athletes, but are practiced by people exercising independently or in a sports/health centre. We compared the rankings of professional athletes with those of individuals who are physically inactive, meaning they do not engage in any physical exercises. Participants were evaluated based on the greatest benefits for 11 health-related indicators: BMI, subjective health, depressed mood, stress, sedentary behavior, vigorous physical activity (VPA), sleep, alcohol consumption, smoking, overeating, and eating breakfast. The healthiest group for each specific indicator received 1 point, while the least healthy group received 22 points. The group with the lowest total score across all 11 indicators was considered the healthiest, while the group with the highest total score was deemed the least healthy.

## Results

### Subjective indicators of health

Subjective health assessment did not differ between the three study groups or between men and women (Table 1). However, depressed mood and stress were higher in individuals who did not exercise than in the professional athlete and E-20 groups (*p* < 0.001). Women in all groups marked a higher frequency of depressed mood and higher stress levels than men (*p* < 0.001).

**Figure 1:**
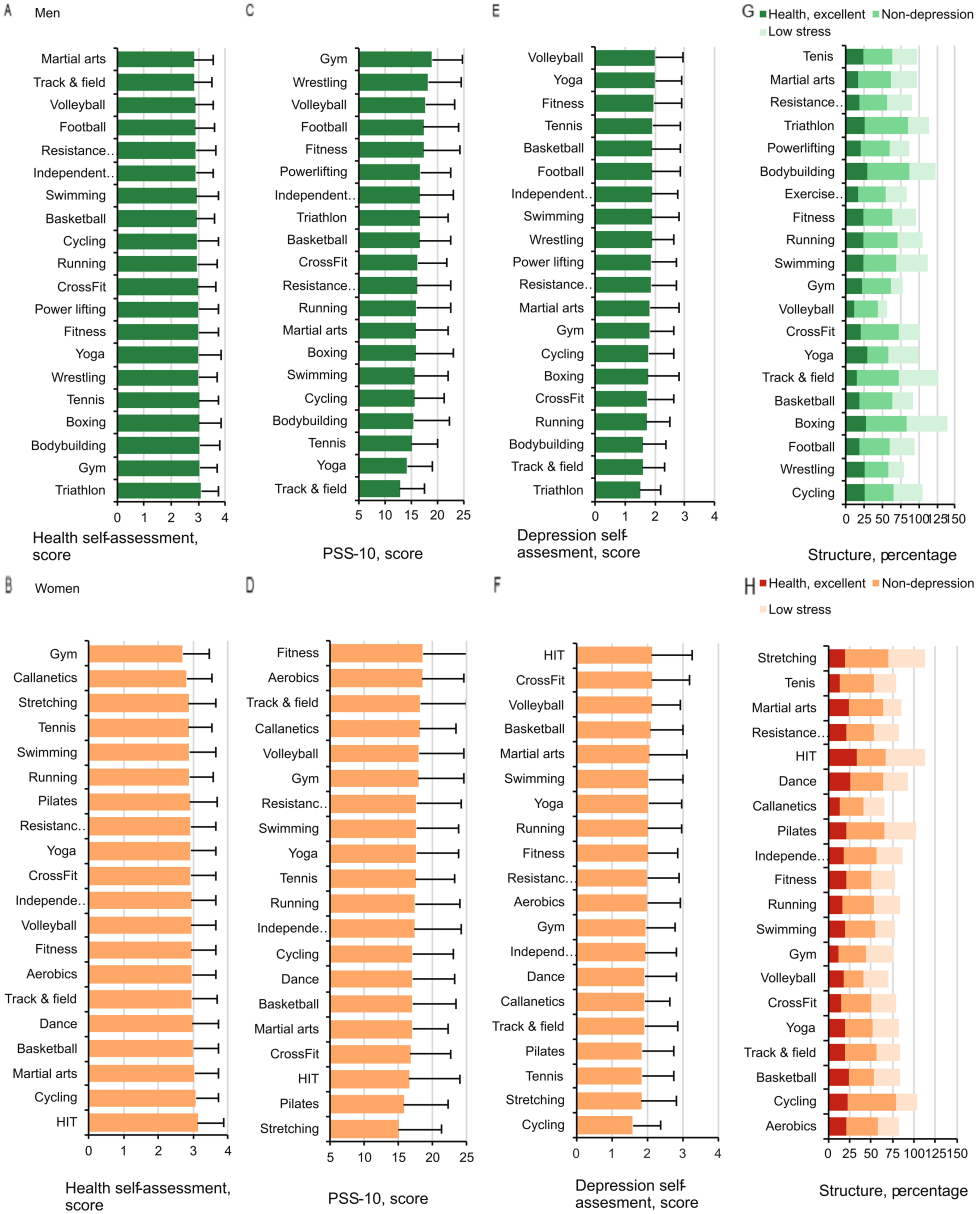
Subjective health, depressed mood, and stress in men **(A,C,E)** and women **(B,D,F)** in the E-20 groups. Structure of subjective health, depressed mood, and stress in men **(G)** and women **(H)**. The data are expressed as the percentages of men and women who rated their health as excellent, did not experience any signs of depressed mood (non-depressed mood), and had the least amount of stress according to participation in the different types of exercise.

The subjective health rating did not differ significantly between women and men in E-20 groups (one-way ANOVA, *p* = 0.94 respectively) (Figures 1A,B). However, 33.3% women in the high-intensity training (HIT) group rated their health as “excellent” compared to 12.5 and 13% in the gym and tennis groups, respectively (*p* < 0.01, between cardio and other groups) (Figures 1G,H). In men, the yoga group had the highest health ranking (29.4%) and the volleyball group had the lowest health ranking (10.4%) (*p* < 0.01).

Within the E-20 groups, the level of depressed mood had significant links to exercise type in both women (*p* = 0.005, ${ŋ}_{P}^{2}$ =0.013) and men (*p* = 0.007, ${ŋ}_{P}^{2}$ =0.033) (Figures 1E,F).

In women, the less frequently depressed mood levels were reported in the cycling group and the highest levels were observed in the HIT, extreme conditioning program training, and volleyball groups (Tukey *post hoc* test, *p* < 0.01). Among men, depressed mood was lowest in the triathlon, track-and-field athletics, and bodybuilding groups, and highest in the volleyball and yoga groups (*p* < 0.01).

Stress level was found to have considerable links to exercise type in both women (*p* < 0.001, ${ŋ}_{P}^{2}$ =0.018) and men (*p* = 0.003, ${ŋ}_{P}^{2}$ =0.035) (Figures 1C,D). In women, the lowest stress level was observed in the stretching group and the highest in the fitness and aerobics groups (*p* < 0.01). In men, stress was lowest in the track-and-field athletics and yoga groups, and highest in the gym, wrestling, and volleyball groups (*p* < 0.01).

### Body mass index and energy expenditure structure

**Figure 2:**
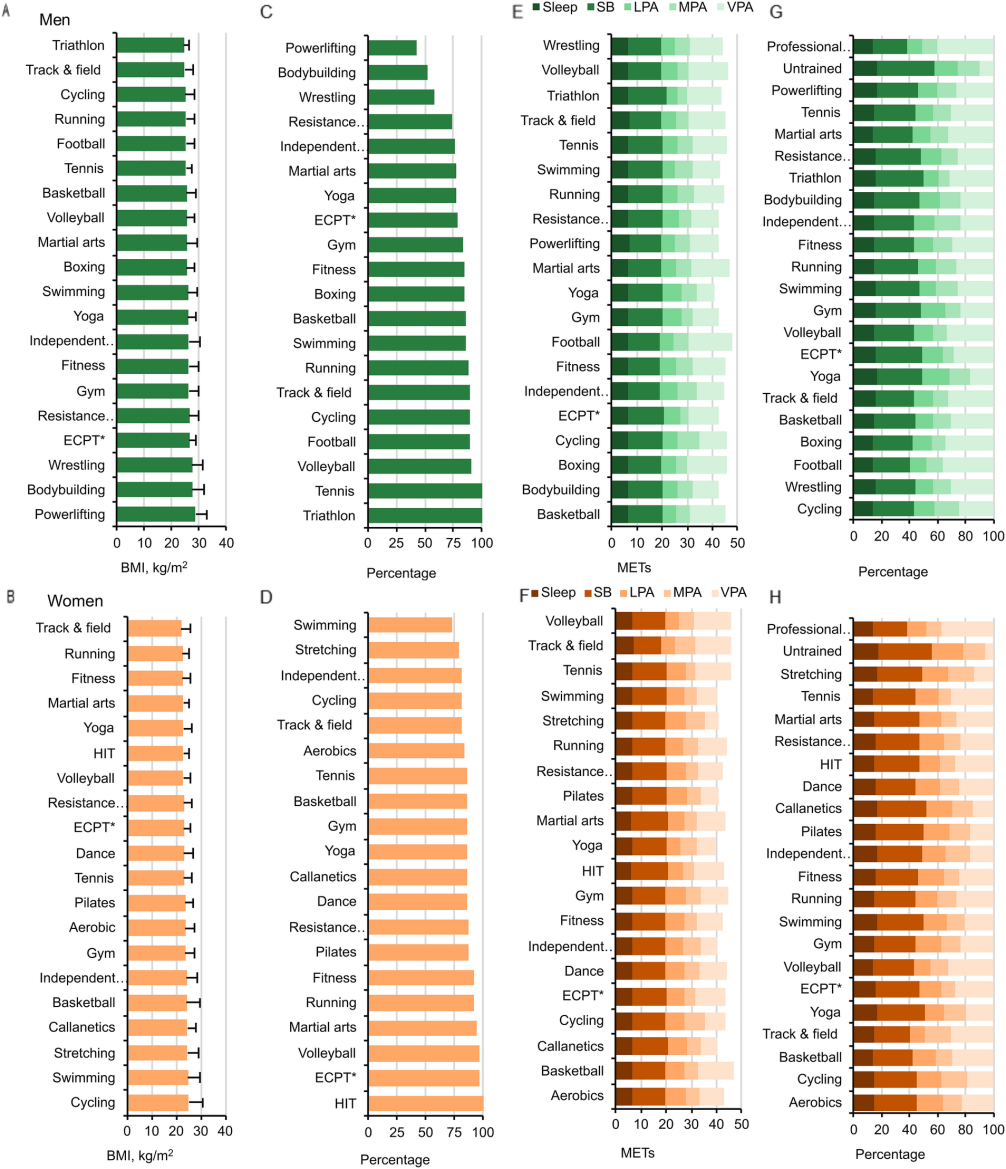
BMI in men **(A,C)** and women **(B,D)**. Data for BMI (kg/m^2^) are shown in the top panels, and for the percentages of participants with a normal BMI (18.5–25 kg/m^2^) in the bottom panels. ECPT*- extreme conditioning program training. Energy expenditure during sleep, SB, LPA, MPA, VPA METs. SB – sedentary behavior, LPA – light-intensity physical activity, MPA – moderate-intensity physical activity, VPA – vigorous-intensity physical activity in men **(E)** and women **(F)**. **(G,H)** Present the respective energy expenditure structure in percentages for men and women.

BMI was found to have considerable links to exercise type in both women (*p* < 0.001, ${ŋ}_{P}^{2}$ =0.048) and men (*p* < 0.001, ${ŋ}_{P}^{2}$ =0.06) (Figures 2A–D). In women, BMI was lowest in the track-and-field athletics and running groups, and highest in the cycling, swimming, and stretching groups (*p* < 0.01). In men in the E-20 groups, BMI was lowest in the triathlon and track-and-field athletics groups, and highest in the bodybuilding, powerlifting, and wrestling groups (*p* < 0.01). Interestingly, all women in the HIT group and all men in the triathlon and tennis groups had a normal BMI.

Sleep METs was considerably linked to exercise type in both men (*p* < 0.001, ${ŋ}_{P}^{2}$ =0.041) and women (*p* = 0.008, ${ŋ}_{P}^{2}$ =0.01) (Figures 2E–H). In women, the longest sleep duration was in the track-and-field athletics group, and the shortest in the HIT and martial arts groups (*p* < 0.01). In men, the longest sleep duration was in the track-and-field athletics and powerlifting groups, and the shortest in the martial arts group (*p* < 0.01).

SB was found to have significant links to exercise type in both women (*p* < 0.001, ${ŋ}_{P}^{2}$ =0.033) and men (*p* < 0.001, ${ŋ}_{P}^{2}$ =0.059). In women, sedentary time was longest in the martial arts and HIT groups, and shortest in the track-and-field athletics groups (*p* < 0.01). In men, sedentary time was longest in the triathlon group and shortest in the powerlifting and track-and-field athletics groups (*p* < 0.01).

LPA, MPA, and VPA were found to have significant links to exercise type in both women (*p* = 0.003, ${ŋ}_{P}^{2}$ =0.014; *p* = 0.011, ${ŋ}_{P}^{2}$ =0.013; *p* < 0.001, ${ŋ}_{P}^{2}$ =0.33, respectively) and men (p = 0.011, ${ŋ}_{P}^{2}$ =0.018; *p* = 0.005, ${ŋ}_{P}^{2}$ =0.034; p < 0.001, ${ŋ}_{P}^{2}$ =0.23, respectively) (Figures 2F–H). In women, VPA was highest in the volleyball, track-and-field athletics, basketball, and tennis groups, and lowest in the stretching, Callanetics, Pilates, and yoga groups (*p* < 0.01). In men, VPA was highest in the football, boxing, volleyball, martial arts, and track-and-field athletics groups, and lowest in the yoga group (*p* < 0.01).

TEE METs was considerably linked to the type of exercise in women (*p* < 0.001, ${ŋ}_{P}^{2}$ =0.13) and in men (*p* < 0.001, ${ŋ}_{P}^{2}$ =0.124). In women, TEE METs was highest in the basketball, volleyball, track-and-field athletics, and tennis groups, and lowest in the stretching, Callanetics, Pilates, and yoga groups (*p* < 0.01). In men, TEE METs was highest in the football, boxing, volleyball, and martial arts groups, and lowest in the yoga group (p < 0.01).

### Smoking, alcohol consumption, and healthy eating

**Figure 3:**
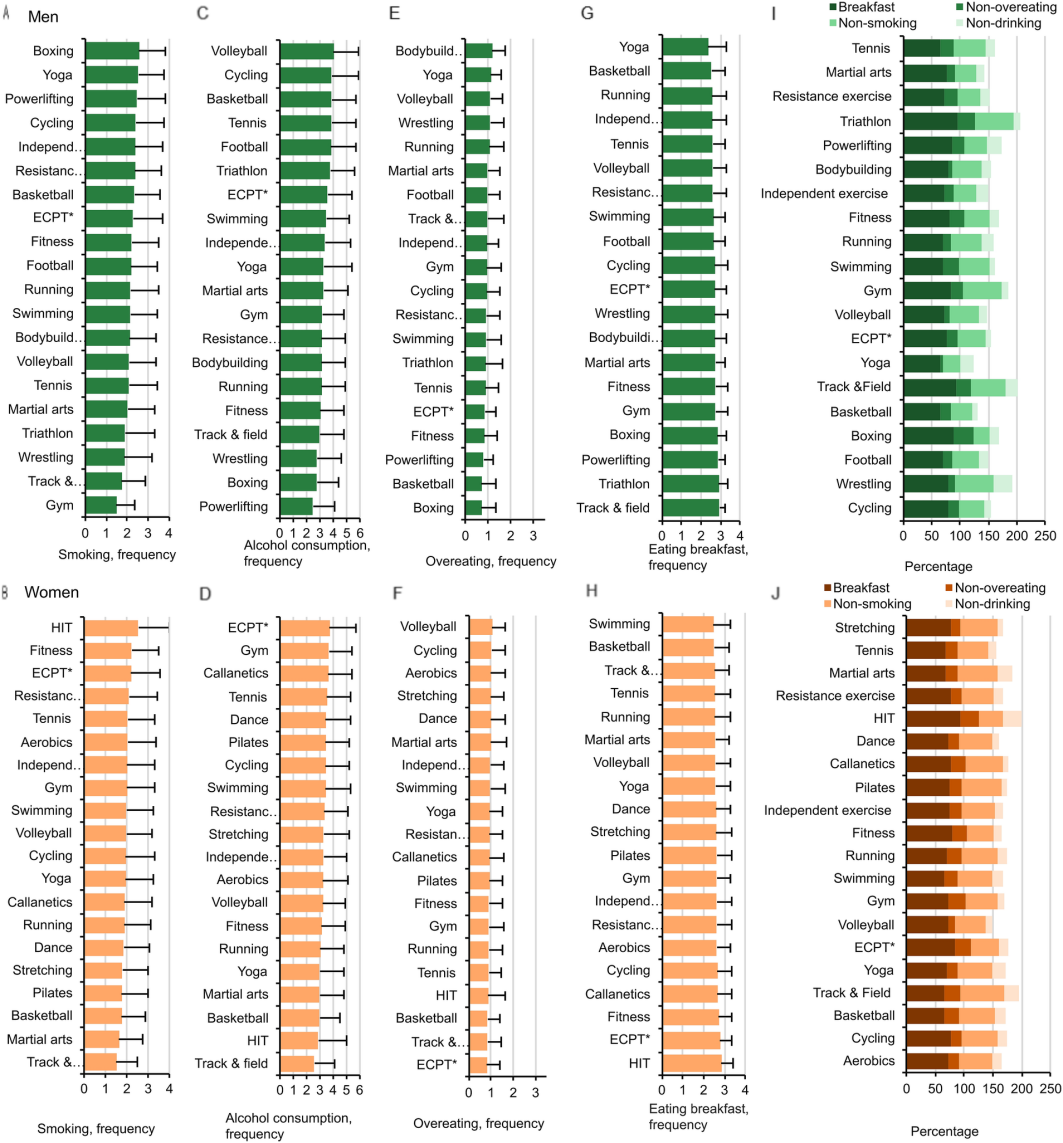
Smoking, alcohol consumption, overeating, and eating breakfast in men **(A,C,E,G)** and women **(B,D,F,H)** in the E-20 groups. Percentages of men **(I)** and women **(J)** who do not smoke (non-smoking), do not consume alcohol (non-drinking), do not overeat (non-overeating), and eat breakfast in women and men in the E-20 groups ECPT*- extreme conditioning program training.

Smoking was related to exercise types in both women (*p* = 0.048, ${ŋ}_{P}^{2}$ =0.011) and men (*p* = 0.003, ${ŋ}_{P}^{2}$ =0.035) (Figures 3A,B). Alcohol consumption was significantly related to exercise type in men (*p* < 0.001, ${ŋ}_{P}^{2}$ =0.055) but not in women (*p* = 0.102, ${ŋ}_{P}^{2}$ =0.011) (Figures 3C,D). Eating breakfast was considerably linked to exercise type in both women (*p* = 0.011, ${ŋ}_{P}^{2}$ =0.013) and men (p < 0.001, ${ŋ}_{P}^{2}$ =0.041) (Figures 3G,H), but overeating was not linked to exercise type in women (*p* = 0.313, ${ŋ}_{P}^{2}$ =0.008) or men (*p* = 0.5, ${ŋ}_{P}^{2}$ =0.019) (Figures 3E,F).

Women in the extreme conditioning program training, track-and-field athletics, basketball, and HIT groups overate the least, and those in the volleyball, cycling, aerobics, and stretching groups overate the most (*p* < 0.01). Men in the basketball and boxing groups overate the least, and those in the bodybuilding, yoga, volleyball, and wrestling groups overate the most (*p* < 0.01). Breakfast was eaten most often in the HIT group in women, and in the track-and-field athletics and triathlon groups in men.

In summary, according to these health behavior components, HIT and track-and- field athletics groups ranked the first among women in the E-20 group, and triathlon and track-and-field athletics groups ranked the first in men (Figures 3I,J).

### General ranking

**Figure 4:**
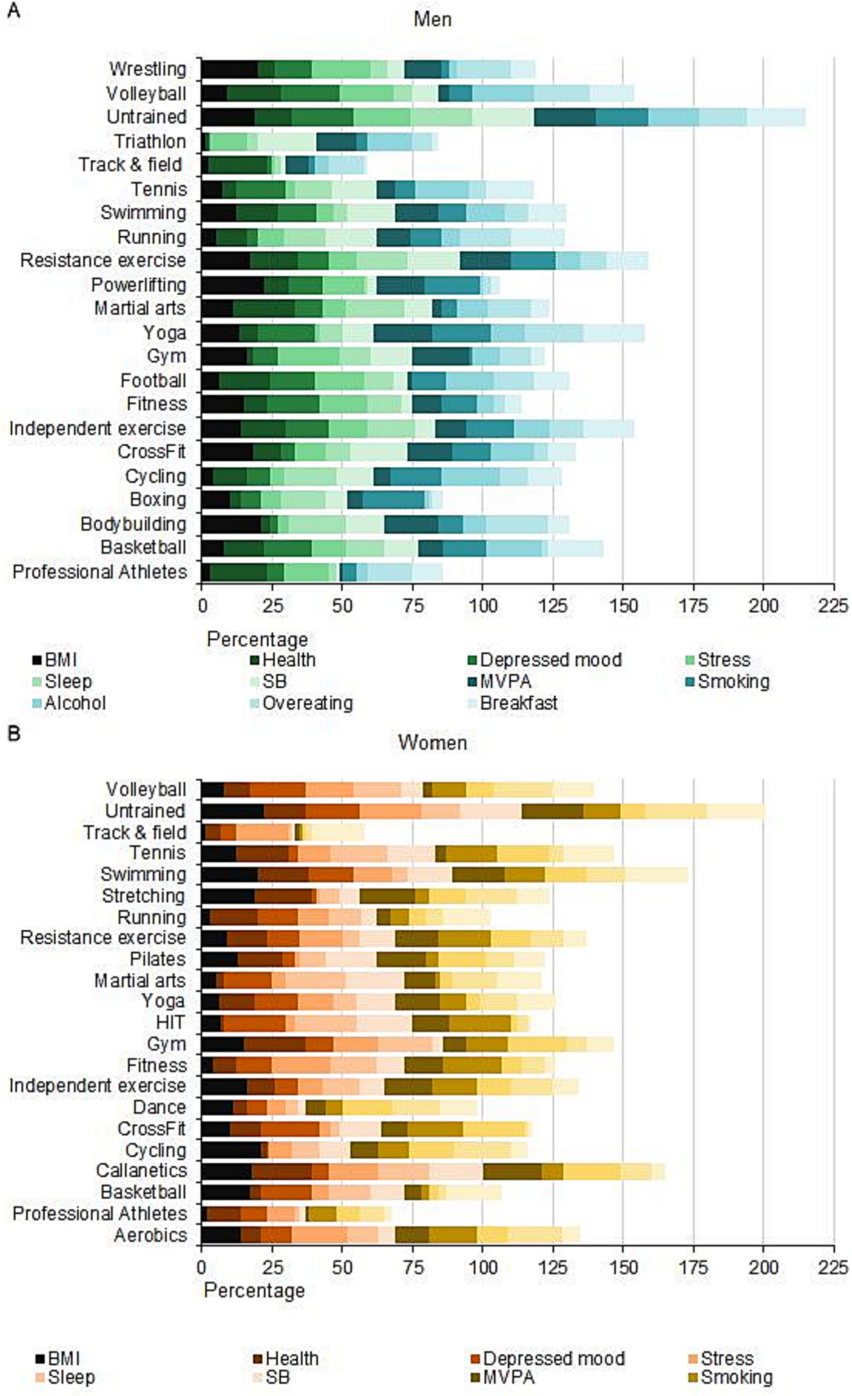
General rankings of men **(A)** and women **(B)** of the E-20 groups and the group that does not exercise (Untrained) ECPT*- extreme conditioning program training.

The summation of the E-20 ranking according to the 11 indicators showed that track-and-field athletics groups ranked the highest (lowest score) in both men and women (Figures 4A,B). The total scores for the 11 indicators were lower in all exercise groups compared with the physically inactive group that does not exercise (“Untrained” in Figure 4). In the E-20 groups, the second and third overall rankings were dance and running for women and triathlon and boxing for men, respectively.

## Discussion

The original finding of our study is that health-related indicators (subjective health, sleep, BMI, SB, LPA, MPA, VPA, MVPA, TEE, stress, smoking, alcohol consumption, depressed mood, eating breakfast, and overeating) are linked with participating in professional sports and with the 20 most popular types of exercise (E-20) performed independently or in a sports/health centre in Lithuania compared with people who do not exercise. This association was found in both men and women, although there were some gendered differences in the patterns related to specific types of exercise.

Our study is unique in that it comprehensively examines a relatively large number (11) of health-related indicators and includes all components of 24-h energy expenditure (sleep, SB, LPA, MPA, and VPA). Additionally, we examined whether these indicators differ between the three populations studied (professional athletes, those who exercise for health, and those who do not exercise), whether they are associated with specific types of the E-20 exercises, and whether these patterns differ between men and women. Other studies described the impact of only a few exercise types on health-related indicators. Activities and specific health parameters that have been studied previously include swimming, fencing, yoga, and body conditioning for enhancing mood and reducing stress (7); walking/running, cycling, and team sports for depression reduction (11); tennis, badminton, soccer, cycling, swimming, jogging, and callisthenics for life expectancy (8); aerobics for depression reduction (26); eight exercise types for mental health (9); ball games and judo for quality of sleep and quality of life (27); yoga for reduction of depression symptoms (28); stair climbing, weight-lifting, walking, stretching, and aerobics for lowering the risk of mortality (29); walking, running, cycling, dance, golf, and stretching to lower all-cause mortality (30); and strength training, walking, and vigorous exercise for changing nutritional behavior (13).

As far as we are aware, our research is the first to indicate that any form of sport or exercise is associated with notable reductions in depressed mood, stress, BMI, sedentary behavior, and overeating in both men and women, and with increases in eating breakfast, VPA, MVPA, TEE, and sleep duration in men. Other studies have shown that exercise reduces the negative effects of stress on human health (31). Interestingly, in our study, smoking and alcohol consumption were not related to specific exercises in women. It was clear from the total scores in our study that the lifestyles of the participants who are professional athletes or who exercise independently or in a sports/health centre were healthier. We found that participation in professional sports was better than E-20 exercising in reducing BMI and SB (compared with all other groups), and increasing sleep duration, VPA, MVPA, and TEE (and even decreasing LPA) in both men and women.

Unexpectedly, we found that the specificity of exercising did not affect the subjective health assessment in both women and men; that is, health was assessed equally by all three groups (professional athletes, participants in E-20, and the untrained). These finding correlate with the findings from other studies reporting that changes in psychological and social factors (including exercise) impact people’s well-being positively over the life-span (32). Indisputably, human physical activity is influenced not by one, but by many factors, and it is hard to distinguish which factors are the most important (33). Our study found it challenging to differentiate the cause(s) from the consequence(s) due to possible links between the two through various interrelationships; that is, low depressed mood or good health is likely to stimulate participation in physical activity, and higher physical activity may improve health and reduce depressed mood. For example, obesity and low physical activity are interrelated in that the lack of physical activity contributes to obesity and obesity lowers the motivation to exercise (34) and, due to the incapability to suppress eating cravings, contributes to more recurrent overeating (35).

In our study, stretching, HIT, cycling, and Pilates ranked best among women in terms of health, stress, and depressed mood. Among men, boxing, bodybuilding, and track-and-field athletics ranked best according to these indicators. Unexpectedly, yoga and games did not rank among the leading exercise types in reducing stress and depression, although yoga (28, 36) and team sports (11) were found to reduce symptoms of depression. Aerobic exercise has been found to reduce depression (26), and we found the less frequently depressed mood among women in the cycling group and among men in the triathlon group.

Breakfast was eaten most regularly by women in the HIT group and the track-and-field athletics groups. Women in the HIT group and men in the boxing and basketball groups overate the least. Healthy eating behavior is influenced by various factors, e.g., physical activity, socio-economic status, social support, gender, and age (37). The lowest alcohol consumption was in the track-and-field athletics and HIT groups in women and in the powerlifting group in men. Women in the track-and-field athletics group and men in the gym and track-and-field athletics groups smoked the least.

The primary limitation of this study is the potential overestimation of physical activity due to the self-reported questionnaire used. Research from Denmark indicates that this scale often inflates reported time spent on vigorous, moderate, and light-intensity physical activities, while it tends to underestimate sedentary time (38). Furthermore, comparing physical activity data across studies is challenging because of the diverse methods used to quantify physical activity (2). Another key limitation is the observational cross-sectional design of the study, which prevents us from establishing causal relationships between physical activity and health indicators. Since we assessed independent and dependent variables simultaneously, we cannot determine the temporal direction of the observed associations. This lack of a temporal framework means that, although we observed differences between physically active and inactive participants, we cannot conclude whether physical activity directly influenced health outcomes or if individuals with better health were simply more likely to engage in physical activity. Additionally, our study did not account for potential confounders, such as diet, socioeconomic status, and genetic predispositions, which could have influenced the observed associations. While we controlled for key demographic variables, the possibility of unmeasured confounding factors remains, potentially affecting the robustness of our findings. Moreover, within the professional athlete group, we could not compare health indicators across different sports disciplines due to an insufficient number of participants. Similarly, we did not analyze the impact of physical exercise across different age groups because we lacked reliable data for such an assessment. Despite these limitations, our study provides valuable insights into the differences in health-related indicators between physically active and inactive individuals.

## Conclusion

Our study indicates that participants who engaged in physical activity generally scored higher on various health-related scales compared to those who were inactive. These benefits include reductions in depressed mood, stress, body mass index (BMI), sedentary behavior (SB), and overeating. Additionally, there are improvements in habits such as eating breakfast regularly, engaging in vigorous physical activity (VPA), participating in moderate-to-vigorous physical activity (MVPA), and total energy expenditure (TEE). Notably, men also experienced improvements in sleep duration. These positive effects were observed in both genders. In women, smoking and alcohol consumption were not linked to the type of exercise performed. Participation in professional sports proved to be more effective than exercise type E-20 in reducing BMI and SB, as well as in increasing sleep duration, VPA, and MVPA for both men and women. Male professional athletes also tended to consume less alcohol and smoke less than individuals in other groups. However, professional sports participation did not lead to greater reductions in depressed mood and stress compared to E-20. The rankings of the 11 health indicators for exercise type E-20 varied between men and women. Nevertheless, the track-and-field athletics group achieved the highest overall health indicator rankings for both genders.

## Data Availability

The original contributions presented in the study are included in the article/supplementary material, further inquiries can be directed to the corresponding author.
